# Supplementary material for: Qualitative Investigation into the Mental Health of Healthcare Workers in Japan during the COVID-19 Pandemic
Source: Int J Environ Res Public Health. 2022 Jan 5;19(1):568. doi: 10.3390/ijerph19010568 (PMC8744919; doi:10.3390/ijerph19010568)
Supplement: Supplementary file 1 [file ijerph-19-00568-s001.zip › ijerph-1477650-supplementary File S1.pdf]

**Qualitative investigation into the mental health of healthcare workers in Japan during the COVID-19 pandemic**

**Supplementary Material: Participants' Comments for Each Theme**

**Theme S1: Increased Stress and Loneliness**

*P1 (Participant 1): There were no established guidelines nor legal support and protection for us. I had to look up many things, and interpret various pieces of information. ... I also had to report our cases to the management. These have significantly increased my workload.*

*P21: There was a sense of loneliness. We used to work together, including colleagues from different departments, but now we are all segmented, and space to be used for treatment is limited.*

*P16: As a healthcare worker, I must not be infected, as that will impact the hospital's reputation. This means that my family also had to restrict their daily behaviours, which was especially stressful for my children.*

*P6: At an early phase, one COVID-19 patient died, and staff at that hospital was criticised. That was very unfair. My team works at that hospital one day a week, so now we are treated like a virus in our hospital. 'You are dangerous because you work there'.*

*P10: Though this is not limited to healthcare workers, there is a discrimination against people who are infected. They are unreasonably criticised [for being infected]. Anyone could be*

*infected. Sometimes it's not within their control. Staff at a care home, where a cluster was found, was and still is criticised.*

*P21: If people know that there is a positive case among staff at the hospital, it will be a big deal. Everyone at the hospital thinks 'I don't want to be the first one'. Stigma for COVID is strong. We had a patient who was infected, but she moved to another town because people in the community were harsh to her.*

**Theme S2: Reduced Strategies for Coping**

*P22: We used to have a lot of chitchats, for example, at the end of our shifts. While writing a daily report, we also talk about how our families are or what we did on a weekend. ... During a shift, sometimes we have to have direct, negative or intense conversations, but chitchats will help retain our relationship: you know that the person doesn't dislike you.*

*P19: As a physiotherapist, what I can do for my patients is now limited as my work usually involves direct touch on the patient's body. I feel less of the meaning of work, hatarakigai, as now I don't feel like I am a physiotherapist sometimes.*

*P13: We cannot travel, meet with friends, and engage with hobbies, so there is no way to destress ourselves. Moreover, we cannot have social gatherings with colleagues, which now I realise, are very important for our wellbeing, knowing each other better.*

*P22: I used to visit my grandma's grave every summer, as she inspired me to be a healthcare worker. ... My wife has been looking forward to visiting my family during the summer, but we haven't been able to.*

**Theme S3: Communication and Acknowledgement as a Mental Health Resource**

*P4: My team communicates well. One reason is probably that our team leader is easy to talk to, and listens to us well.*

*P3: It is very helpful to connect with healthcare workers who are in a similar circumstance to me. ... This kind of conversation happens organically in the face-to-face context, but now we need a video call to do that.*

*P10: I feel comfortable talking about my mental distress with my colleagues.... Everyone is available and willing to help, if I need to talk.*

*P5: Positive feedback from my line manager or the head of the hospital helps me cope with stress. Also, some patients brought me some gifts, appreciating my treatment. That kind of moment is helpful for my mental health.*

*P8: My line manager and the hospital head are very supportive of my work that has been expanded due to COVID. They are also supportive of other activities such as applying for a grant. ... Their acknowledgement means a lot to me.*

*P20: Today many healthcare workers who work with COVID patients are featured in a TV programme or a section in news shows. Because of that, people's understanding towards those workers has been increasing. But those who don't directly work with COVID patients are also impacted. They want to be acknowledged too. In many cases, just a 'thank you' would be enough.*

**Theme S4: Understanding of Self-Care**

*P4: My line manager believes that if we don't take good care of ourselves, we cannot take care of others. He supports self-care, which positively impacts our workplace culture. I am very thankful to him for that.*

*P7: My team endorses self-care, it may be because we are in palliative care. If I think about the culture among doctors in Japan, I don't think that's the case.*

*P8: Now we cannot go anywhere for a vacation nor get together with friends. Mentally it is very hard. ... Japanese healthcare needs to shift from their traditional value system, and embrace self-care and mental health care more.*

*P9: Self-care is important but when a patient visits you, you have to attend to them. It's in the law. Management team, often older generations, advises doctors to stay at the hospital for long hours so that they can see more patients. But younger generations want to have a good balance between work and life; they want to engage with their family life too.*

*P16: I think it's in Japanese culture. We cannot say we are suffering, or we are in pain, because other people may be also … Japanese people are not good at taking care of themselves. That is a taboo, you cannot say that in this culture.*

*P8: As a doctor, I find it hard to self-care. ... The root of this is a value of Japanese people during WWII, 'We don't ask for anything until we win'. We believe that asking for something means we are not cooperating. But in reality, we need to care for ourselves, before care for others. ... It's been a challenge for me to take good care of myself.*
